# Intraluminal Migration of Surgical Sponge: Gossypiboma

**DOI:** 10.4103/1319-3767.65195

**Published:** 2010-07

**Authors:** Kundan K. Patil, Shaifali K. Patil, Kedar P. Gorad, Anuradha H. Panchal, Sahil S. Arora, Raj P. Gautam

**Affiliations:** Department of Surgery, MGM, Medical College, Kamothe, Navi Mumbai, India; 1 Department of Obstetrics and Gynaecology, MGM, Kalamboli, India

**Keywords:** Gossypiboma, intraluminal migration, intestinal obstruction

## Abstract

Surgical mop retained in the abdominal cavity following surgery is a serious but avoidable complication. The condition may manifest either as an exudative inflammatory reaction with formation of abscess, or aseptically with a fibrotic reaction developing into a mass. Intraluminal migration is relatively rare. We report the case of a 23 year old woman who presented after a previous caesarean section with intestinal obstruction. Plain abdominal radiograph and computed tomography confirmed the presence of gossypiboma. The patient underwent laparatomy and sponge removal. This report discusses the approach to, and manifestations of, migratory surgical gossypiboma.

Surgical mop retained in the abdominal cavity following surgery, is a serious but avoidable complication. Gossypiboma, term derived from the Latin “gossypium” (cotton) and the Swahilli “boma” (place of concealment)[1] is the term for retained surgical sponge. Two usual responses to retained mops are exudative inflammatory reaction with formation of abscess, or aseptic with fibrotic reaction to develop a mass;[2] intraluminal migration is relatively rare, leading to obstruction. Patients develop symptoms of abdominal pain, nausea, vomiting, anorexia, and weight loss resulting from obstruction or a malabsorption type syndrome caused by the multiple intestinal fistulas or intraluminal bacterial overgrowth.[1] Early recognition of this entity will ensure prompt institution of appropriate treatment, reducing morbidity and mortality in such patients.

## CASE REPORT

**Figure 1:**
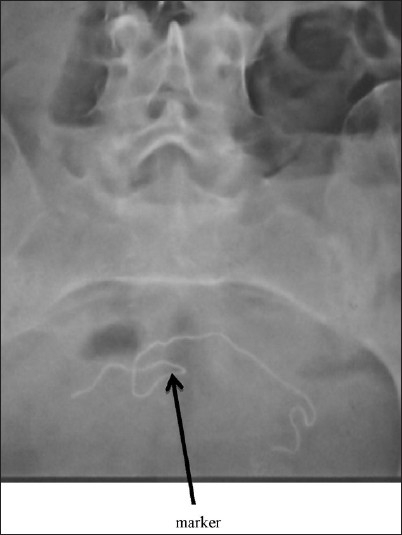
X-ray showing intestinal obstruction with marker of mop

A 23 year old woman presented with a history of caesarean section done three months ago at a private hospital and was admitted to our hospital. She repeatedly complained of colicky pain in left iliac fossa since two weeks, vomiting and constipation since 10 days. On physical examination her lower abdomen was tender and bowel sounds were hyperactive. Rectal examination was normal. Plain abdominal radiograph revealed multiple air fluid shadows in ileum and jejunum with a linear radio-opaque foreign body in lower abdomen which raised suspicion of retained surgical sponge [Figure 1].

**Figure 2:**
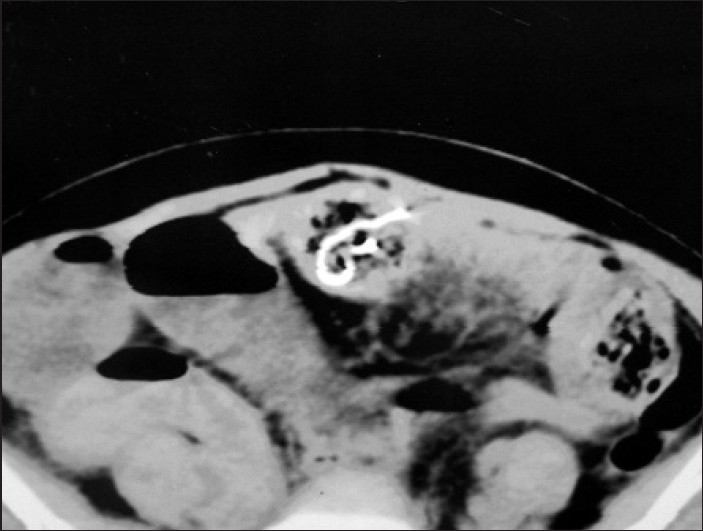
CT scan showing marker of mop

A computed tomography (CT scan) of the abdomen was performed using oral and intravenous contrast, revealing an intraluminal foreign body with tiny air bubbles containing metallic marker suggestive of gossypiboma [Figure 2].

**Figure 3:**
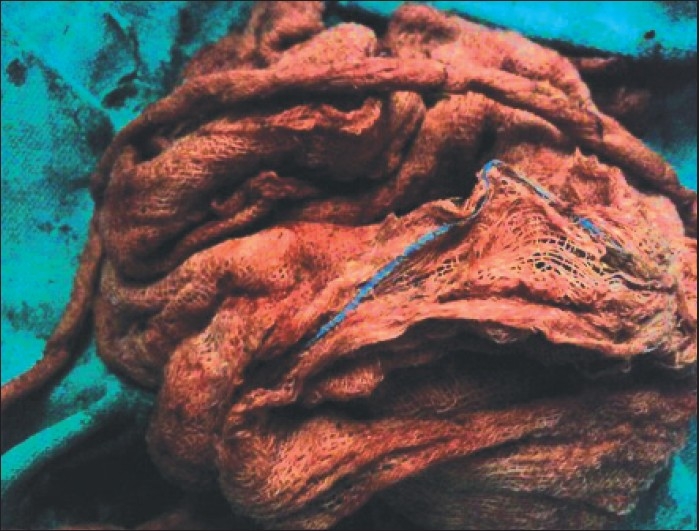
Removed intraluminal mop

Exploratory laparatomy was performed. There were no lesions in peritoneal cavity or perforation or fistula or adhesion. On palpation of small bowel, a mass was felt in the distal ileum 10 centimeters proximal to ileocaecal junction. Intestine and mesentry were inflamed and edematous. Proximal enterotomy was done to extrude the mop [Figure 3]. Enterotomy was closed in two layers. Post-operatively, the patient recovered uneventfully. As we did not have experts in laparoscopy at that time, laparotomy was done.

## DISCUSSION

Retained surgical sponge occurs at a frequency of one per 100-3000 operations.[3] The possibility of a retained foreign body should be in the differential diagnosis of any postoperative patient who presents with pain, infection, or palpable mass. Pathologically, a retained sponge may lead to foreign body reactions of two types- formation of foreign body granuloma due to aseptic fibrinous response, or exudative reaction leading to abcess formation.[4] Migration of retained sponge into bowel is rare compared to abcess formation and occurs as a result of inflammation in the intestinal wall that evolves to necrosis.[5] The intestinal loop closes after complete migration of sponge.[6]

Peristaltic activity advanced the mop usually to stay in the terminal ileum, resulting in obstruction.[5,7] As no fistulous tract was identified it is difficult to explain the course of events leading to intraluminal migration. The CT findings of a sponge usually describe a rounded mass with a dense central part and an enhancing wall. Other features of retained sponges or towels include a whorl-like appearance with trapped air bubbles and cystic masses with infolded densities. The three most significant risk factors are emergency surgery, unplanned change in the operation, and body mass index.

Prevention of gossypiboma can be done by simple precaution like keeping a thorough pack count and tagging the packs with markers. New technologies are being developed which will hopefully decrease the incidence of retained foreign body. An electronic article surveillance system which uses a tagged surgical sponge that can be identified electronically has been examined.[8] Bar codes can be applied to all sponges, and with the use of a bar code scanner the sponges can be counted on the back table.[1] The low index of suspicion is due to rarity of condition and latency in the manifestation of symptoms. It frequently results in misdiagnosis, leading to delay in proper management. If diagnosis is made early, laparoscopic retrieval may be feasible.[9]
